# Regression of severe sclerotic chronic graft-versus-host disease after a second haploidentical hematopoietic stem cell transplantation: a case report

**DOI:** 10.3389/fmed.2026.1929283

**Published:** 2026-09-25

**Authors:** Rongmu Luo, Qihang Man, Xiaomei Zhang, Hexian Guan, Wenjing Gu, Jingbo Wang

**Affiliations:** 1 Department of Hematology, Aerospace Center Hospital, Beijing, China; 2 Department of Hematology, China Aerospace Science & Industry Corporation 731 Hospital, Beijing, China

**Keywords:** fibrosis, graft vs. host disease, salvage therapy, scleroderma, localized, transplantation, haploidentical

## Abstract

Sclerotic chronic graft-versus-host disease (Scl-cGVHD) is a disabling fibrotic manifestation of cGVHD and frequently exhibits poor responsiveness to conventional immunosuppressive therapy. Therapeutic options remained limited, particularly in pediatric patients. An 8-year-old boy with relapsed acute myeloid leukemia underwent matched sibling donor allogeneic hematopoietic stem cell transplantation (allo-HSCT) in January 2021. Four months later, he developed progressive cutaneous cGVHD that evolved into severe Scl-cGVHD, with diffuse skin thickening, painful fissures, ulceration, and multiple joint contractures, loss of independent ambulation, and complete dependence in activities of daily living. Skin biopsy demonstrated extensive dermal fibrosis, collagen deposition, and increased *α*-smooth muscle actin (*α*-SMA) expression. Despite multiple lines of immunosuppressive and targeted therapy, including corticosteroids, calcineurin inhibitors, mycophenolate mofetil, ruxolitinib, rituximab, and other agents, the disease progressed, with the modified Rodnan skin score (mRSS) reaching 35. Because of refractory disease and disabling disease, a second haploidentical HSCT from his father was performed in September 2022. Following transplantation, disease progression ceased and gradual clinical improvement occurred. At 12 months, the mRSS decreased from 35 to 2, with substantial recovery of skin flexibility and joint mobility. Repeat skin biopsy showed reduced collagen compactness and markedly decreased interstitial *α*-SMA expression, consistent with attenuation and remodeling of dermal fibrosis. Serial immune monitoring revealed progressive recovery of B- and NK-cell compartments and sustained T-cell reconstitution after the second transplantation, in contrast to prolonged B-cell depletion and limited NK-cell recovery after the first transplant. At 24 months, complete donor chimerism persisted, leukemia remained in remission, the mRSS reached 0, and the patient regained independent walking and activities of daily living. This case describes sustained clinical regression and histopathological attenuation of severe, established Scl-cGVHD following a second haploidentical HSCT. The temporal association with immune reconstitution raises the possibility that restoration of immune homeostasis may have contributed to disease remodeling. However, causality cannot be established from a single case. Given the substantial risks associated with second allo-HSCT, this approach should not be considered standard therapy and may be considered only in exceptional, highly selected patients after multidisciplinary risk-benefit assessment.

## Introduction

Chronic graft-versus-host disease (cGVHD) develops in approximately in 40%–50% of patients undergoing allogeneic hematopoietic stem cell transplantation (allo-HSCT) and remains a major contributor to late non-relapse mortality and long-term morbidity (1, 2). It represents a complex immune-mediated disorder involving elements of alloimmunity, autoimmunity, dysregulated B-cell activation, impaired immune tolerance, and progressive tissue fibrosis (3). The skin is one of the most frequently affected organs. Among cutaneous manifestations, the sclerotic phenotype particularly debilitating, which occurs in about 13% of patients with cGVHD (4, 5). Sclerotic cGVHD (Scl-cGVHD) is characterized by progressive dermal and fascial fibrosis, skin tightening, edema, joint contractures, and neuropathic pain (6, 7). These changes can severely restrict mobility and daily functioning, leading to substantial and often irreversible disability (8).

Although post-transplant cyclophosphamide (PTCy)-based prophylaxis has reduced the incidence of Scl-cGVHD to 5.8% at 5 years after transplantation, established disease remains difficult to control once it develops (9). In practice, Scl-cGVHD is frequently resistant to standard immunosuppressive treatment (10). More recently, several targeted agents, including ibrutinib, belumosudil, and ruxolitinib, have been approved for cGVHD and have improved outcomes in steroid-refractory cases (11–13). However, complete responses remain uncommon, particularly in patients with established sclerotic disease (11, 14). As a result, truly effective treatment options for advanced Scl-cGVHD remain limited.

Here, we present a pediatric case of severe, treatment-refractory Scl-cGVHD following matched sibling donor (MSD) transplantation. After failure of multiple therapeutic lines, the patient underwent a second haploidentical HSCT and achieved durable clinical remission. The objective of this report is to describe a novel salvage strategy for treatment-refractory Scl-cGVHD, and to discuss the potential immunological rationale and substantial risks associated with this approach. This case report adheres to the CARE (CAse REport) reporting guidelines, and the completed checklist is provided as Supplementary Material.

## Case presentation

### Patient information

An 8-year-old boy with relapsed acute myeloid leukemia (AML) was referred for allo-HSCT. There was no relevant family history of autoimmune or fibrotic diseases. The patient previously achieved remission after induction chemotherapy but relapsed within one year, prompting consideration of allogeneic transplantation.

### Clinical findings and diagnostic assessment

On January 21, 2021, the patient underwent allo-HSCT from his human leukocyte antigen (HLA)-identical sibling donor. The sibling donor was clinically healthy at the time of donation. During approximately five years of follow-up after donation, the donor remained free of autoimmune diseases, chronic inflammatory disorders, abnormal immune manifestations, or hematological abnormalities. The conditioning regimen consisted of total body irradiation (TBI, 400 cGy daily for 3 days), cladribine (9 mg/m^2^ for 5 days), and cytarabine (2.0 g/m^2^ for 5 days). GVHD prophylaxis included cyclosporine A (trough 150-200 ng/mL from day +5), mycophenolate mofetil (MMF, 15 mg/kg twice daily from day +5), and PTCy administered on day +3 (50 mg/kg) and day +4 (30 mg/kg). Neutrophil and platelet engraftment were uneventful. Due to the development of cGVHD, cyclosporine A was switched to tacrolimus, and MMF was continued because of persistent GVHD activity.

The patient did not develop acute GVHD after the first transplantation. Four months post-transplant, the patient developed cGVHD predominantly affecting the skin and deep fascia, leading to extensive sclerosis, joint contractures, and severe functional impairment the patient developed cutaneous cGVHD with progressive induration and reduced mobility. No clinically significant involvement of other cGVHD target organs, including the liver, gastrointestinal tract, lungs, eyes, or oral mucosa, was observed. Over subsequent months, the disease evolved into diffuse Scl-cGVHD. Physical examination revealed marked skin tightening of the trunk and extremities, painful fissures, recurrent ulcerations, and contractures of multiple large and small joints (Figure 1A). The patient became unable to walk independently and required full assistance for daily activities. The modified Rodnan skin score (mRSS) was 35, indicating severe and extensive skin involvement. The Eastern Cooperative Oncology Group Performance Status (ECOG PS) score was 4. Immune reconstruction after the first MSD transplantation showed a fluctuating CD4+/CD8 + ratio, reflecting persistent immune instability (Figure 1B, red lines). More notably, CD19+ B cells were recurrently undetectable at multiple time points between months 6 and 15 (Figure 1B, red lines).

**Figure 1:**
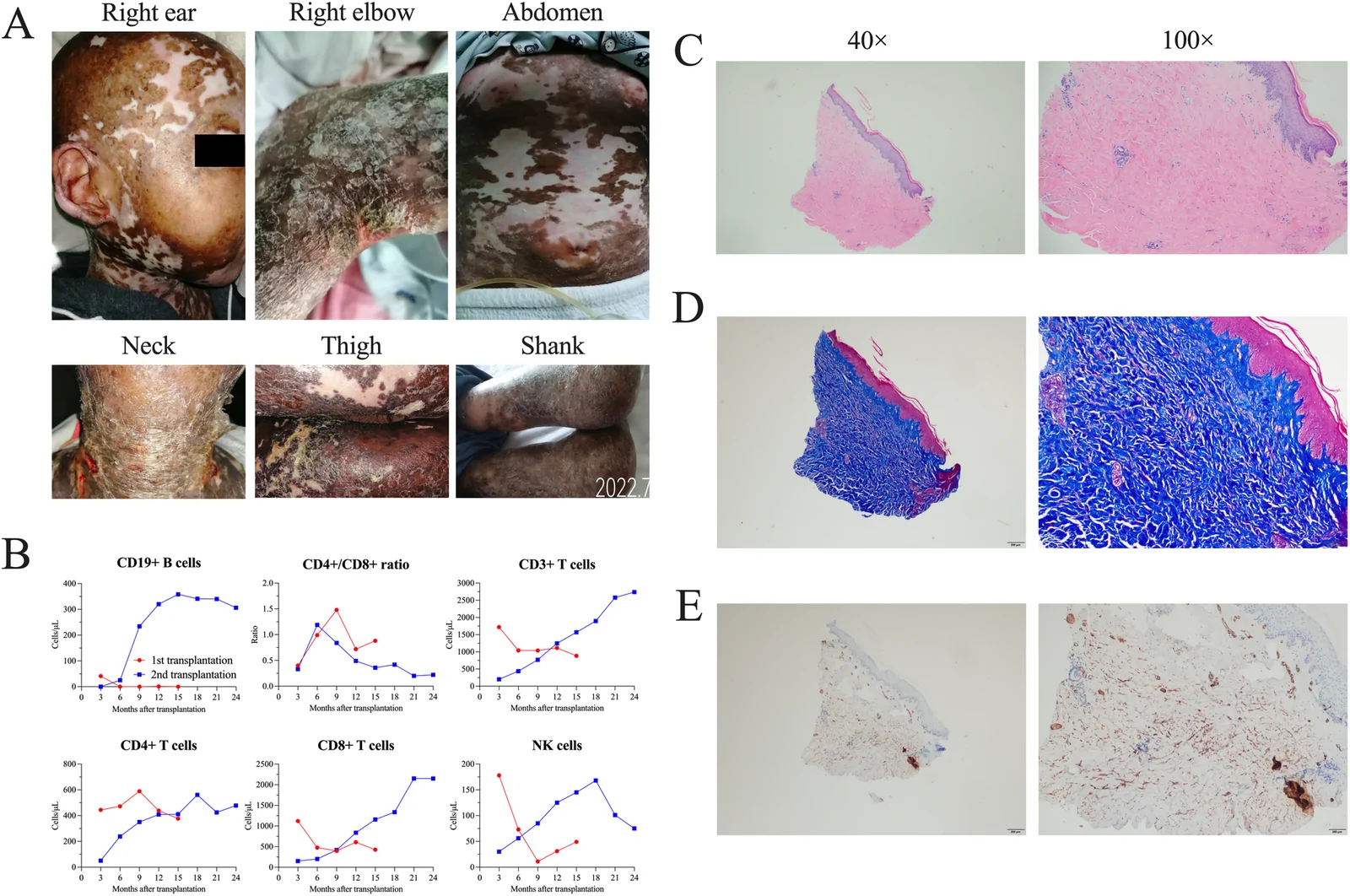
Diffuse Scl-cGVHD in an 8-year-old boy after a MSD transplantation. **(A)** Clinical photographs showing extensive skin thickening and sclerosis, fissures, and joint contractures. **(B)** Immune reconstruction after transplantation. Red lines: the first transplantation; blue lines: the second transplantation. **(C)** Hematoxylin and eosin staining of abdominal skin biopsy showing dermal thickening with compact eosinophilic collagen bundles and loss of normal dermal architecture, without epidermal abnormalities. **(D)** Masson's trichrome staining showing extensive dermal collagen deposition and disorganized collagen architecture. **(E)** *α*-SMA immunohistochemical staining demonstrating abundant *α*-SMA-positive interstitial cells consistent with activated myofibroblasts. *α*-SMA: *α*-smooth muscle actin; MSD: matched sibling donor; Scl-cGVHD: sclerotic chronic graft-versus-host disease.

Hematoxylin and eosin (H&E) staining of skin biopsy from the abdomen demonstrated dermal thickening with dense eosinophilic collagen bundles replacing the normal loose dermal architecture (Figure 1C). Collagen fibers appeared compact and closely packed with reduced interstitial spaces, consistent with sclerotic skin involvement. Masson's trichrome staining further demonstrated extensive collagen accumulation with densely packed and disorganized collagen bundles throughout the dermis (Figure 1D). Immunohistochemical staining for *α*-smooth muscle actin (*α*-SMA) revealed abundant *α*-SMA-positive spindle-shaped interstitial cells in addition to vascular staining, suggesting increased myofibroblast activation during active fibrosis (Figure 1E).

### Therapeutic interventions

After cGVHD onset, first-line therapy comprised tacrolimus (trough 6-10 ng/mL), MMF (0.5 g every 12 h), and prednisone (2 mg/kg/day). Because of progression, second-line and third-line agents were sequentially administered, including rituximab (375 mg/m^2^ weekly for 4 weeks), ruxolitinib (10 mg twice daily for 3 months), etanercept (25 mg weekly for 4 weeks), umbilical cord-derived mesenchymal stem cells (1 × 10⁶/kg weekly for 4 weeks), anti-CD25 monoclonal antibody (1 mg/kg on days 1, 3, 8, 15), and azathioprine (50 mg twice daily for 2 months). These combined therapies still failed to stop disease progression. Cyclophosphamide was then given (50 mg/kg for 2 days, followed by 50 mg/day for 3 months), but skin fibrosis and contractures continued to progress.

### Second haploidentical HSCT

Despite multiple lines of systemic therapy, the patient had severe, progressive Scl-cGVHD, with extensive skin and fascial involvement, profound functional impairment, and life-threatening complications including recurrent infections and bleeding from fissures, A decision to proceed with a second allo-HSCT was made after multidisciplinary discussion. The potential benefits of disease control were weighed against the substantial risks associated with a second allo-HSCT, including treatment-related toxicity, infection, graft failure, recurrent GVHD, and non-relapse mortality. Available therapeutic alternatives were also reviewed. Given the progressive and disabling nature of the disease, the failure or limited accessibility of alternative therapies, and the patient's hematologic status (complete remission with MRD negativity), the multidisciplinary team and family considered second transplantation only as an exceptional salvage approach.

The second allo-HSCT from the patient's father was performed on September 16, 2022. The donor-recipient pair was sex-matched (male to male). HLA mismatch was at 5/10 loci. ABO blood group was major matched (donor A+, recipient A+). The stem source was granulocyte colony-stimulating factor (G-CSF)-mobilized peripheral blood, with a total infused mononuclear cell (MNC) dose of 10.3 × 10^8^/kg, CD34 + cell dose of 11.4 × 10^6^/kg, and CD3 + cell dose of 9.85 × 10^8^/kg. The conditioning regimen was modified myeloablative, consisting of busulfan (0.8 mg/kg every 6 h for 3 days) and cyclophosphamide (50 mg/kg/day for 2 days). GVHD prophylaxis followed the ‘Beijing Protocol’ (15), including anti-thymocyte globulin (ATG, 2.5 mg/kg/day from day −5 to −2), cyclosporine A (trough 150–200 ng/mL from day −1), short-course methotrexate (15 mg/m^2^ day +1; 10 mg/m^2^ days +3, +6, +11), and MMF (15 mg/kg twice daily from day −1). Cyclosporine A was maintained at a target trough concentration of 100–200 ng/mL during the first year and subsequently reduced 50–100 ng/mL during the second years. It was gradually tapered according to clinical status, although low-dose cyclosporine A (25 mg every 12 h) was still being administered at 24 months after transplantation. MMF was given until day +28.

### Follow-up and outcomes

Neutrophil engraftment occurred on day +11, and platelet engraftment on day +18. Bone marrow chimerism using short tandem repeat-polymerase chain reaction (STR-PCR) showed 100% donor type on day +28, sustained thereafter. The patient did not develop acute GVHD post-second transplant. Mild liver cGVHD occurred approximately four months after transplantation and was successfully controlled by increasing the cyclosporine A dosage for one week. No progression or involvement of other organs was observed thereafter. A mild intestinal infection on day +6 resolved with antibiotics. Minimal residual disease (MRD) monitoring for AML, performed at 2, 6, and 12 months post-transplant, was negative at all time points.

Following the second HSCT, progression of Scl-cGVHD ceased. Over the first year, skin softening and joint mobility gradually improved (Figure 2A). At 12 months, the patient regained full elbow and finger extension, normal cervical rotation, and marked functional recovery (Figure 2B). The mRSS dropped from 35 to 2 (Figure 2C). Serial immune reconstitution after the second haploidentical transplant demonstrated a progressive and sustained recovery of T cell subsets (Figure 1B, blue lines). CD19+ B cells, although undetectable at month 3, showed a surge between month 6 and 9 and sustained stable onward (Figure 1B, blue lines).

**Figure 2:**
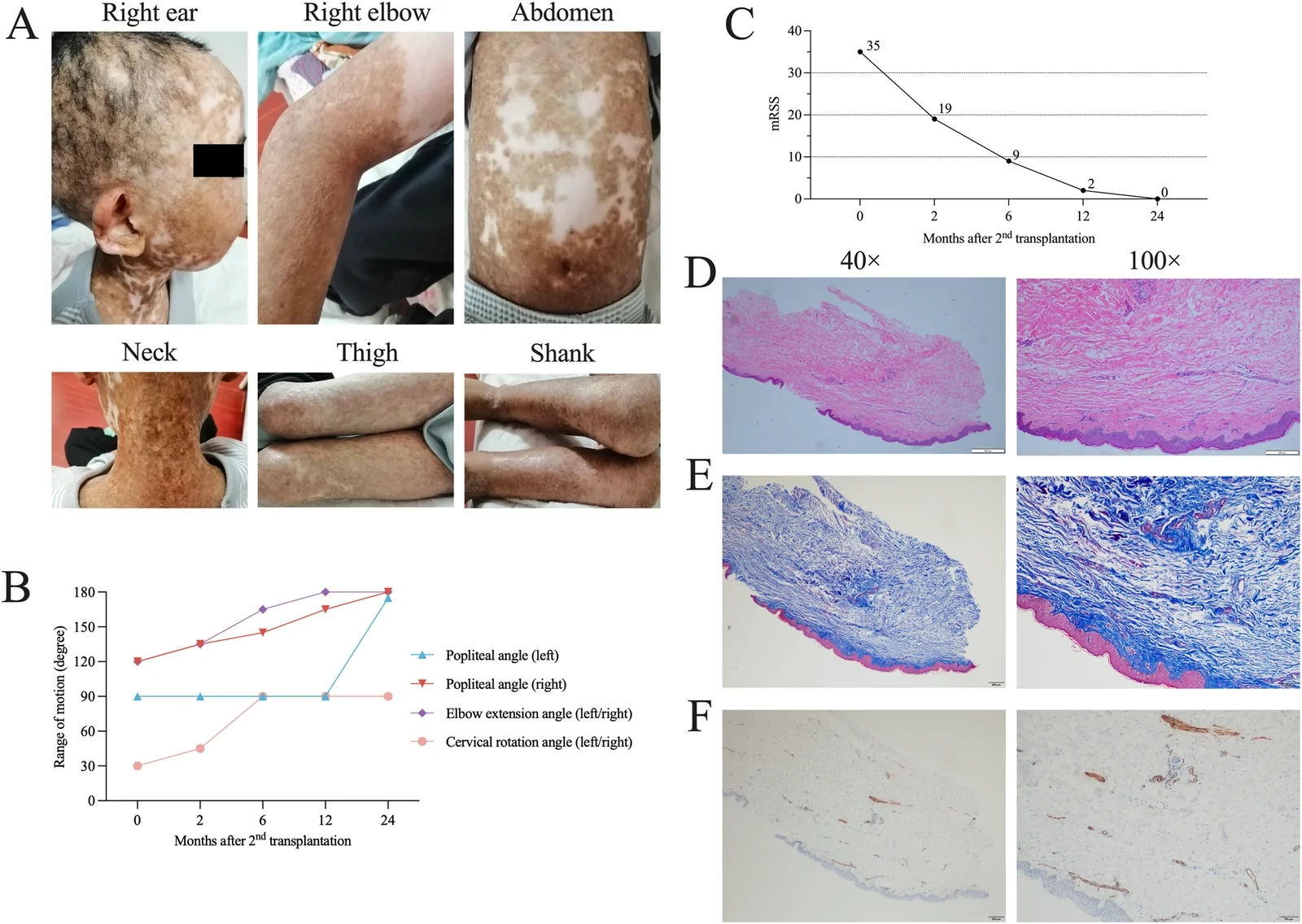
Clinical and histopathological improvement at one year following second haploidentical transplantation. **(A)** Clinical photographs showing gradual softening of the skin and improvement in joint mobility, with healing of fissures and ulcers. **(B)** Quantitative improvement of multiple joint angles (elbow extension, finger extension, cervical rotation). **(C)** Longitudinal decline in mRSS from 35 pre-second transplant to 2 at one year post-transplant. **(D)** Hematoxylin and eosin staining of skin biopsy showing no significant fibrotic tissue proliferation in the dermis or subcutaneous layers. **(E)** Masson's trichrome staining showing partial remodeling of dermal collagen architecture with reduced compactness and improved organization. **(F)** *α*-SMA immunohistochemical staining demonstrating markedly decreased interstitial *α*-SMA expression with residual positivity mainly confined to vascular structures. *α*-SMA: *α*-smooth muscle actin; mRSS: modified Rodnan skin score; Scl-cGVHD: sclerotic chronic graft-versus-host disease.

H&E staining of repeat skin biopsy from the same site as the first biopsy showed restoration of dermal architecture with less compact collagen arrangement and increased interstitial spaces (Figure 2D). Masson staining demonstrated that bundles appeared more organized and less compact, indicating remodeling of the extracellular matrix and attenuation of dermal fibrosis (Figure 2E). Although residual collagen deposition remained detectable, overall dermal fibrosis appeared attenuated. Concurrently, *α*-SMA immunostaining showed a marked reduction in interstitial *α*-SMA-positive cells, with residual staining predominantly confined to vascular structures (Figure 2F).

During the second year, further improvement was noted, including softer skin and continued recovery of joint mobility (Figure 3). At 24 months, mRSS decreased to 0, indicating substantial regression of skin sclerosis. ECOG PS improved to 1. Residual pigmentation changes persisted, including hypopigmentation and hyperpigmentation, but without functional impairment. The patient resumed independent ambulation and normal daily functioning after rehabilitation exercise. A comprehensive timeline of disease onset, treatments, and clinical milestones is provided in Figure 4.

**Figure 3:**
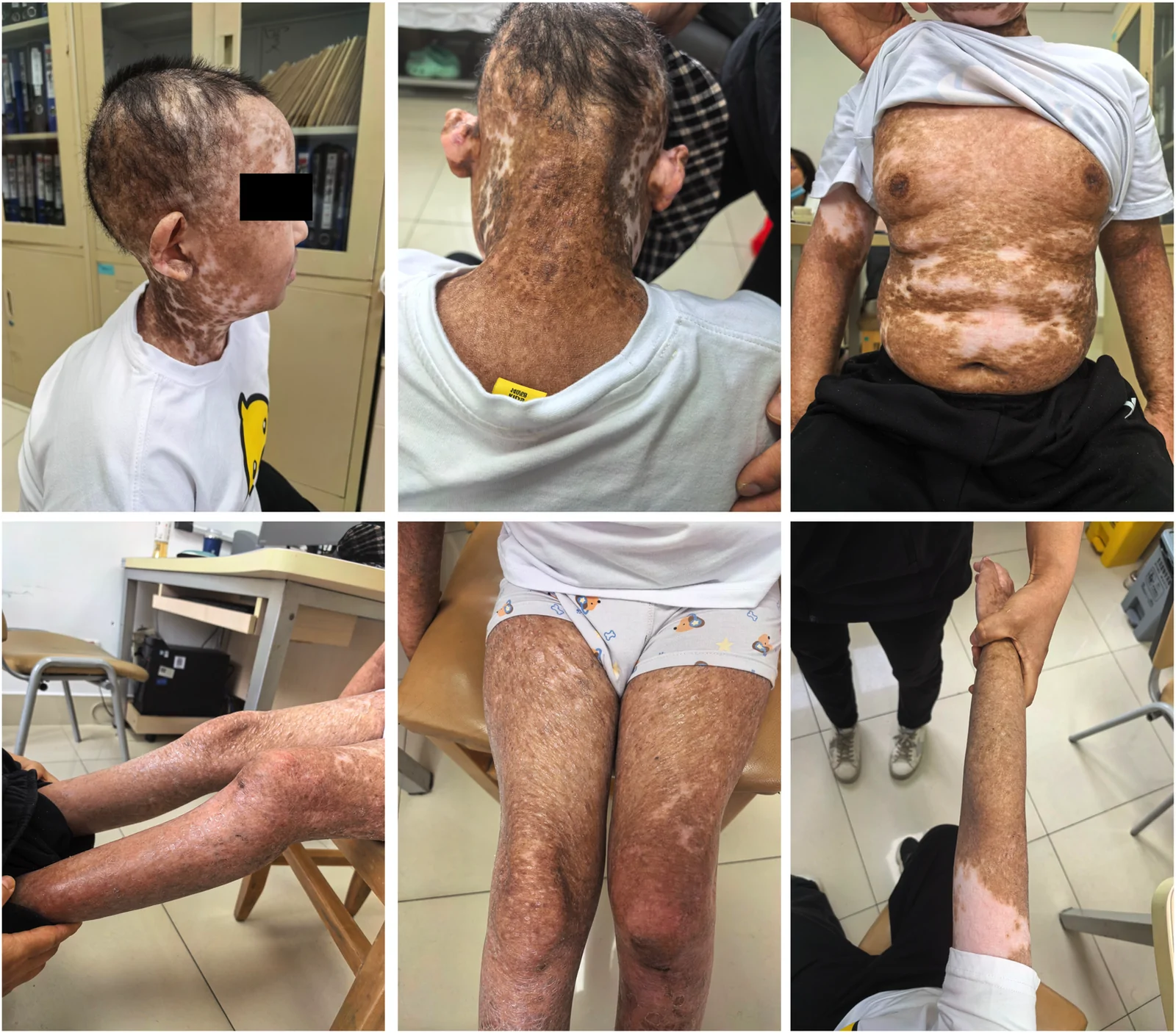
Substantial regression of skin sclerosis with persistent residual pigmentation changes at 2 years after second haploidentical transplantation. Clinical photographs demonstrate near-normal skin texture and joint mobility. The mRSS decreased to 0, indicating substantial resolution of clinical sclerosis. Residual hypopigmentation and hyperpigmentation are visible but without functional impairment.

**Figure 4:**
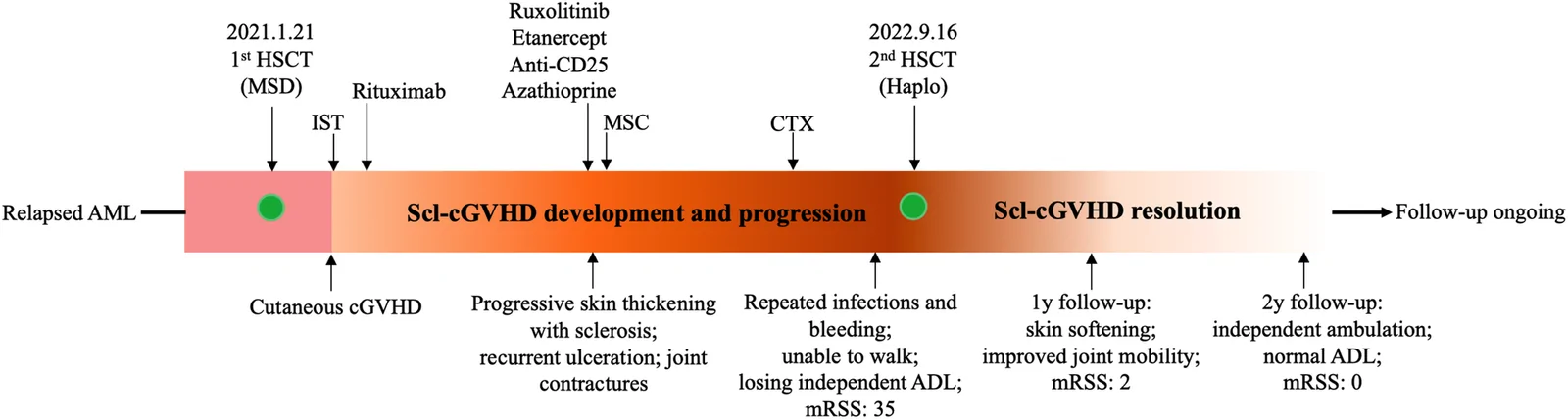
Comprehensive timeline of Scl-cGVHD occurrence, progression, therapeutic interventions, and clinical resolution. Key clinical milestones include first MSD-HSCT (January 2021), onset of Scl-cGVHD (May 2021), failure of multiple systemic therapies (2021-2022), second haploidentical HSCT (September 2022), progressive clinical improvement (2022-2024), and substantial regression of skin sclerosis. ADL: activities of daily living; AML: acute myeloid leukemia; cGVHD: chronic graft-versus-host disease; CTX: cyclophosphamide; HSCT: hematopoietic stem cell transplantation; IST: immunosuppressive therapy; mRSS: modified Rodnan skin score; MSC: mesenchymal stem cells; MSD: matched sibling donor;; Scl-cGVHD: sclerotic cGVHD.

### Patient perspective

The family reported that the patient's progressive loss of mobility and independence was devastating, with profound negative impact on quality of life and psychological well-being. The child was forced to suspend schooling. The patient and parents described the gradual skin softening and regained ability to walk and write after the second transplant as dramatic and life-changing. The boy is now preparing to return to school. They hope that sharing their experience may help other children facing similar untreatable complications.

## Discussion

This case report describes an unusual course of severe, treatment-refractory Scl-cGVHD that developed after MSD-HSCT and subsequently underwent sustained clinical regression following a second haploidentical HSCT. The temporal association is noteworthy, particularly given the severity and chronicity of the sclerotic disease and the accompanying histopathological changes. However, this single observation does not establish that the second transplantation itself caused the improvement. The contribution of the conditioning regimen, changes in concomitant immunosuppression, rehabilitation, delayed effects of previous therapies, supportive care, and the natural evolution of the disease cannot be completely excluded. Therefore, the present case should be regarded as a hypothesis-generating clinical observation rather than evidence of treatment efficacy.

Disparity at loci within the major histocompatibility complex (MHC) remains a central determinant of alloreactive immune responses after allo-HSCT (16). Even in the context of a MSD transplant, cGVHD may occur, most commonly attributed to immune recognition of minor histocompatibility antigens (MiHAs) (17–19). Such responses can persist despite full HLA identity and may contribute to long-term immune activation following transplantation. The longitudinal immune reconstitution findings provide a potentially informative but associative observation. After the first MSD-HSCT, the patient exhibited prolonged depletion of circulating B cells, limited NK-cell recovery, and an unstable T-cell compartment during the period in which severe Scl-cGVHD developed and progressed. In contrast, following the second haploidentical HSCT, progressive recovery of B cells and NK cells occurred together with sustained T-cell reconstitution and coincided temporally with gradual clinical improvement. The increase in circulating B cells between 6 and 9 months after the second HSCT was particularly notable, as delayed B-cell reconstitution and impaired B-cell homeostasis have previously been associated with active cGVHD (20–25, 53). These observations raise the possibility that restoration of immune homeostasis may have accompanied, or potentially contributed to, disease remodeling. However, these observations do not establish that immune reconstitution caused disease regression. No detailed immunophenotyping, cytokine profiling, T- or B-cell functional assays, antigen-specific immune analyses, or donor-specific antibody studies were performed. Moreover, the second transplantation differed from the first in donor relationship, conditioning regimen, GVHD prophylaxis, and other aspects of post-transplant management, making it impossible to attribute the subsequent clinical course to any single immunological factor. Thus, the immune reconstitution data should be regarded as hypothesis-generating and may provide a rationale for future mechanistic studies rather than evidence of a causal immune mechanism.

An especially interesting paradox in this case is that the second transplant used a haploidentical donor, historically considered a higher immunologic risk platform than MSD transplantation, yet it was followed by clinical improvement rather than worsening cGVHD. In a previous analysis of allo-HSCT recipients, use of an HLA-mismatched donor was independently associated with a lower risk of Scl-cGVHD compared with an HLA-matched donor (6). Experimental studies showed that responses to MiHAs were not generated in the presence of stimulation by mismatched MHC antigens after HLA-mismatched transplantation, suggesting that HLA mismatch may exert an immunodominant effect that suppresses alloreactive T-cell responses against MiHAs (26). However, given the absence of antigen-specific immune profiling in our case, the mechanism regarding the immunodominant effect is speculative and hypothesis-generating, intended to guide future research rather than draw causal conclusions.

The patient received multiple systemic therapies, including rituximab and ruxolitinib, for steroid-refractory cGVHD (SR-cGVHD). However, these treatments failed to halt progression of the sclerotic disease, highlighting the refractory nature of established Scl-cGVHD. Rituximab, a monoclonal anti-CD20 antibody targeting B cells, demonstrated an overall response rate of approximately 70% in SR-cGVHD, with particular benefit in cutaneous and musculoskeletal involvement (27, 28). Ruxolitinib, a Janus kinase (JAK)1/2 inhibitor targeting inflammatory cytokine signaling pathways, has shown stronger efficacy in SR-cGVHD. In the phase III REACH3 trial, ruxolitinib achieved a significantly higher overall response rate than best available therapy (49.7% vs. 25.6% at week 24), along with improved failure-free survival (13). A meta-analysis also reported high response rates in skin involvement (29).

Other novel agents for SR-cGVHD, including ibrutinib, a Bruton's tyrosine kinase (BTK) inhibitor targeting B-cell receptor signaling (12, 30), and belumosudil, a Rho-associated coiled-coil-containing protein kinase 2 (ROCK2) inhibitor (31, 32), were not prescribed for this patient. At the time of the second transplantation (September 2022), ibrutinib had been approved in China for the treatment of cGVHD. Belumosudil had not yet been marketed in China and was therefore unavailable. Extracorporeal photopheresis (ECP) had also not been approved in China, and the patient could not access this therapy (33, 34).

Despite encouraging responses overall, an important issue is that most clinical trials of these agents evaluated global cGVHD rather than specifically sclerotic phenotypes (35). Standard outcome measures, including the National Institutes of Health (NIH) Consensus criteria (2, 36), combine multiple organ systems such as skin, liver, gastrointestinal tract, lung, and mucosal involvement into composite response scores. While this framework is appropriate for systemic assessment, it may obscure organ-specific resistance, particularly deep dermal and fascial fibrosis typical of Scl-cGVHD. The NIH skin score significantly relies on body surface area (BSA) estimation and subjective descriptors of “movable” versus “fixed” sclerosis, which may dilute the sensitivity for detecting meaningful changes in pure sclerosis and result in limited sensitivity to changes in sclerotic lesions specifically (37). The NIH Consensus Skin Task Force has recently proposed revisions that separate epidermal and sclerotic components and introduce more objective biomechanical measures (37). Since our case was dominated by severe sclerotic manifestation, we selected mRSS to longitudinally assess the severity of skin involvement. mRSS grades skin thickness across 17 anatomical sites on a scale from 0 (normal) to 3 (severe thickening), with a total score ranging from 0 to 51 (38). It has been previously applied for skin involvement assessment in Scl-cGVHD (39). In our case, serial assessments were performed by the same two experienced physicians who were not blinded to the treatment throughout follow-up. Any disagreement between these two evaluators was resolved by consensus discussion. Thus, serial mRSS measurements allowed us to objectively track the progression and treatment-related changes of skin fibrosis, which was the primary clinical endpoint of interest. Another issue in previous trials is that patients with skin involvement in these trials may include both non-sclerotic and sclerotic disease. Data specific to patients with established fibrosis, as seen in our patient, remain limited.

Very few studies have focused exclusively on SCl-cGVHD. A single-center retrospective study evaluated ruxolitinib in 12 patients with severe Scl-cGVHD (39). Median mRSS decreased from 24 at baseline to 19 at month 3 and to 13 at month 6, but no complete response was observed. In a randomized trial comparing rituximab with imatinib in Scl-cGVHD, only 27% of patients receiving rituximab achieved significant clinical response, and 14% had treatment success (40). In a recent single-arm trial involving 47 patients with refractory Scl-cGVHD, 47% achieved partial response in skin and/or joints after ruxolitinib, but only three patients had complete response in joints and fascia (11). These findings indicate that these approved agents may have limited effectiveness in severe Scl-cGVHD cases.

However, the rapid and profound regression of established sclerosis (mRSS decreasing from 35 to 2 within the first year) observed in this patient deserves particular consideration. Although advanced Scl-cGVHD has traditionally been regarded as a condition in which fibrosis may become irreversible, accumulating evidence suggests that fibrotic remodeling remains a dynamic process involving persistent immune activation, fibroblast stimulation, and extracellular matrix turnover (41, 42). In this pediatric patient, several factors may have contributed to the extensive recovery. First, the enhanced regenerative capacity and tissue plasticity during childhood may have facilitated the fibrosis remodeling (43). Second, the second transplantation introduced a distinct donor-derived immune system and was followed by restoration of B-cell and NK-cell compartments, potentially reducing persistent profibrotic immune stimulation (44, 45). Third, early control of ongoing immune activation may have prevented further fibroblast activation and allowed gradual extracellular matrix remodeling (42). However, these possibilities remain speculative, and the contribution of age-related regenerative capacity versus immune reprogramming cannot be determined from a single case.

Despite the favorable outcome, this case should not be interpreted as evidence that second transplantation is a standard treatment for refractory Scl-cGVHD. Although HSCT is a curative therapy in many medical conditions (46, 47), a second allo-HSCT carries substantial risk, including increased non-relapse mortality (48–50). Previous studies examined second haploidentical transplantation for relapsed hematologic malignancies after a first HLA-matched transplant (49, 51, 52), but resolution of cGVHD was not assessed. As a radical approach, second transplantation for severe refractory Scl-GVHD requires careful risk-benefit evaluation. Future analyses of patients undergoing second transplantation with pre-existing severe cGVHD may clarify how often disease regression occurs and which factors predict success.

Several limitations should be acknowledged. First, as a single case report, causality cannot be established. Second, the absence of mechanistic immunological data, such as immunophenotyping, cytokine profiling, donor-specific antibodies and chimerism-based mechanistic analyses, precludes any causal conclusions about the mechanisms of disease regression. Third, the skin involvement was only assessed by mRSS, a tool that has not been validated in pediatric Scl-cGVHD. Whereas, the NIH cGVHD scoring has not been conducted. Fourth, we could not completely exclude the delayed effect from prior therapies, the conditioning regimen, supportive care, rehabilitation, or the natural course of disease, despite the temporal association with the second transplantation. Further clinical studies, incorporating detailed immunophenotyping, cytokine profiling, and functional T- and B-cell assays, are required to verify the effectiveness of a second transplantation in Scl-cGVHD remission and to elucidate the biological basis of Scl-cGVHD resolution in similar clinical contexts.

## Conclusion

In summary, this case describes sustained clinical regression and histopathological attenuation of severe, treatment-refractory Scl-cGVHD following a second haploidentical HSCT. The temporal relationship raises the possibility that restoration of immune homeostasis may have contributed to disease remodeling. The observed regression raised the possibility that established Scl-cGVDH may retain a degree of reversibility or biological plasticity. However, these hypotheses cannot be established from a single case. Second all-HSCT carries substantial morbidity and mortality and should not be considered standard treatment for refractory Scl-cGVHD. In exceptional cases in which approved and evidence-based therapies have been exhausted, second transplantation may be considered only after careful multidisciplinary risk-benefit assessment and informed discussion with the patient and family.

## Data Availability

The original contributions presented in the study are included in the article/Supplementary Material, further inquiries can be directed to the corresponding author.
